# PKCα Is Recruited to* Staphylococcus aureus-*Containing Phagosomes and Impairs Bacterial Replication by Inhibition of Autophagy

**DOI:** 10.3389/fimmu.2021.662987

**Published:** 2021-03-18

**Authors:** Maria Celeste Gauron, Alexandra C. Newton, María Isabel Colombo

**Affiliations:** ^1^Laboratorio de Mecanismos Moleculares Implicados en el Tráfico Vesicular y la Autofagia-Instituto de Histología y Embriología (IHEM)- Universidad Nacional de Cuyo, CONICET- Facultad de Ciencias Médicas, Mendoza, Argentina; ^2^Department of Pharmacology, University of California San Diego, La Jolla, CA, United States

**Keywords:** *Staphylococcus aureus*, autophagy, xenophagy, Protein Kinase C, LC3

## Abstract

Hijacking the autophagic machinery is a key mechanism through which invasive pathogens such as *Staphylococcus aureus* replicate in their host cells. We have previously demonstrated that the bacteria replicate in phagosomes labeled with the autophagic protein LC3, before escaping to the cytoplasm. Here, we show that the Ca^2+^-dependent PKCα binds to *S. aureus*-containing phagosomes and that α-hemolysin, secreted by *S. aureus*, promotes this recruitment of PKCα to phagosomal membranes. Interestingly, the presence of PKCα prevents the association of the autophagic protein LC3. Live cell imaging experiments using the PKC activity reporter CKAR reveal that treatment of cells with *S. aureus* culture supernatants containing staphylococcal secreted factors transiently activates PKC. Functional studies reveal that overexpression of PKCα causes a marked inhibition of bacterial replication. Taken together, our data identify enhancing PKCα activity as a potential approach to inhibit *S. aureus* replication in mammalian cells.

## Introduction

Autophagy is a cellular degradative process that not only plays an essential role in cellular homeostasis, but also in clearing infection by certain pathogens, a process known as xenophagy. Specifically, xenophagy is a selective defense mechanism by which, once microorganisms are recognized by the cell, proteins known as autophagic adaptors/receptors recruit LC3 and components of the autophagic machinery toward them (1–4). Pathogens sequestered in the interior of autophagosomes are degraded by the fusion of these vacuoles with lysosomes. Many invasive bacterial pathogens, including species of *Salmonella, Shigella* and *Mycobacterium*, induce an autophagic response in host cells, which leads to the degradation of these pathogens or partial restriction of their intracellular growth (5–7). However, the generated autophagic response is occasionally unsuccessful, as some microorganisms are able to manipulate this pathway for their own benefit in order to survive and replicate in the host cells (8). A clear example of this behavior is *Staphylococcus aureus*, a major hospital- and community-acquired pathogen associated with significant mortality (9). Understanding the mechanism by which *S. aureus* evades xenophagy has important therapeutic potential, particularly given the appearance of strains that manifest antibiotic resistance (10).

*S. aureus* exerts its effects both by the release of toxins and enzymes secreted by the bacterium, and by efficiently invading epithelial and endothelial cells (11–13). Once in the interior of the cells, *S. aureus* transits the phagosomal pathway, avoiding lysosomal degradation, to finally escape from phagosomes *via* a toxin-dependent mechanism to further replicate in the cytoplasm of the host cells (14, 15). It has been previously demonstrated that the *S. aureus*-containing phagosome is clearly marked by the autophagic protein LC3 (16). We have previously shown that, although *S. aureus* can localize into autophagosomes, their maturation is blocked and the fusion with lysosomes is inhibited, allowing bacterial replication inside this vacuole (16). Indeed, these *S. aureus*-containing compartments were also identified as autophagosomes by electron microscopy, due to their characteristic double membranes (17). Finally, *S. aureus* escapes from the autophagosomes and once in the cytoplasm, the bacterium induces apoptosis through a caspase-independent mechanism which allows the infection to spread (17).

*S. aureus* infection is accompanied by changes in the intracellular levels of second messengers. For example, it has been reported that when this pathogen invades cells, cAMP levels are decreased (18), leading to reduced activation of the protein Epac, in turn causing, higher levels of autophagy which are beneficial for the staphylococcal infection (19). On the other hand, it has also been reported that *S. aureus* invasion increases intracellular Ca^2+^ levels (20), suggesting that transducers of Ca^2+^ signals might also be involved in the regulation of *S. aureus*-induced autophagy.

A key transducer of Ca^2+^ signals is the subfamily of conventional isozymes of the Protein Kinase C (PKC) family of Ser/Thr kinases. PKC isozymes are involved in transduction of a wide range of extracellular signals that control important cellular functions, including the autophagic response (21). Conventional (PKCα, PKCβ and PKCγ) and novel (PKCδ, PKCϵ, PKCη and PKCθ) PKC family members are activated following receptor-mediated generation of diacylglycerol (DAG), which binds to their diacylglycerol-sensing C1 domains (22). Whereas novel PKC isozymes are activated by DAG alone, the conventional PKC isozymes have a lower affinity for DAG and require Ca^2+^-dependent targeting to membranes *via* their Ca^2+^-sensing C2 domain (22). It has been described that novel PKC isozymes participate both in xenophagy and in host cell responses against bacterial infection. For example, PKCϵ is implicated in innate immunity due to its role in the activation of macrophages in defense against *S. aureus* and *E. coli* infection (23). Also, it has been shown that both DAG and PKCδ have a role in the autophagy of *Salmonella typhimurium* (24). As for Ca^2+^-regulated conventional PKCs, PKCα impairs intracellular replication of *Legionella pneumophila* in macrophages (25), in addition, PKCα has been shown to be important in phagosomal maturation of latex beads phagosomes (26). However, the role of conventional PKC isozymes in xenophagy remains to be elucidated.

In the present report we have determined that DAG is present in the membranes of phagosomes containing *S. aureus* and that a conventional PKC isozyme, PKCα, is able to associate with these compartments in an α-hemolysin dependent manner. We have determined that the presence of PKCα in the *S. aureus* phagosome depends on Ca^2+^ concentration but is independent of the presence of DAG on the phagosomal membranes. Interestingly, in those phagosomes labelled by PKCα, the recruitment of the autophagic protein LC3 was hampered, indicating that the association of both proteins with the phagosomal membrane was mutually exclusive. In addition, we have found that overexpression of PKCα impaired the efficient intracellular replication of the bacteria. In summary, we present evidence that PKCα modulates the autophagic response induced by *S. aureus* in epithelial cells.

## Materials And Methods

### Materials

Cell culture media α-MEM was purchased from Gibco (Invitrogen, Argentina) and fetal bovine serum (FBS) (A15-101) was obtained from GE Healthcare Argentina S.A. Luria-Bertani (LB) broth and agar (Miller) were purchased from Merck (Merck S.A., Buenos Aires, Argentina). Chloramphenicol, gentamycin, U73122, 1-Butanol, Gö6976, PDBu (Phorbol 12,13-dibutirate) and *Staphylococcus aureus* α-hemolysin (H9395) were obtained from Sigma Aldrich. DNA markers Hoechst 33342 and Topro were from Molecular Probes (Buenos Aires, Argentina).

### Cell Culture

Adherent epithelial CHO-K1 (Chinese Hamster Ovary) cells from ATCC were cultured in α-MEM supplemented with 10% of FBS, streptomycin (50μg/ml) and penicillin (50 U/ml).

### Plasmids and Transfection

GFP-PKCα, GFP-PKCβII, GFP-PKCγ, and GFP-PKCη were provided by Dr. Yusuf Hannun (Medical University of South Carolina, USA), GFP-PKCδ and GFP-PKCϵ were a gift from Dr. Dominique Joubert (Universtités Montpellier, Francia). PKCδ-C1-GFP was kindly provided by Dr. Mauricio Terebiznik (University of Toronto, Canada). Cells were transfected with Lipofectamine 3000, Lipofectamine 2000 or JetPrime, following the manufacturer’s instructions.

### Bacteria Strains, Culture, and Infection

*S. aureus* wild type, wt (8325–4), the mutant deficient for α-hemolysin: Hla (–) (DU1090) or the mutant Hla (–) complemented with an α-hemolysin plasmid (DU1090/pDU1212): Hla (–)+pHla were used. All strains were kindly provided by Dr. Claudia Sola (CBICI-CONICET, Córdoba, Argentina) and built by Dr. Richard J. O’Callaghan. Bacterial strains were cultured overnight at 37°C in 10 ml of a LB broth with the proper antibiotics for strain selection: streptomycin for the selection of the plasmid DU1090 and chloramphenicol for the selection of the plasmid pDU1212. For infection experiments, cells were resuspended in infection media (α-MEM supplemented with 10% FBS) to an OD=650 nm of 0.4 (~4x108 CFU) and diluted to a 10:1 multiplicity of infection (MOI). 1 hour post infection, the media was washed to eliminate extracellular bacteria and fresh infection media was added. Afterward, cells were incubated for an additional 3 hours at 37°C, washed with PBS, fixed with 4% paraformaldehyde, and processed for analysis by confocal microscopy.

### Confocal Microscopy and Image Processing

Cells were analyzed with the microscope Olympus Confocal FV1000. The program FV10-ASW 3.0 was used for image acquisition and imaging configuration. The obtained images were processed by the deconvolution tool in ImageJ.

### Real-Time Kinase Activity Monitoring

CHO-K1 cells co-transfected with mCherry-PKCα and CKAR2 (C Kinase Activity Reporter) were maintained in HBSS solution (ThermoFischer) during the experiment. 1 ml of LB broth was added to settle a baseline. After 5 minutes *S. aureus* culture supernatants were added and, after 10 minutes, 1 μM of the PKC inhibitor Gö6976 was added. To study the effect of α-hemolysin, 10 μg/ml or 30 μg/ml of pure protein was added to the cells, followed by 200 nM of the PKC activator PDBu and finally 1 μM Gö6976. Images were acquired using the microscope Zeiss Axiovert (CarlZeiss Microimaging, Inc.) with the digital camera MicroMax (Roper-Princeton Instruments) controlled by the software Metafluor (Universal Imaging, Corp.). FRET (Föster Resonance Energy Transfer) and CFP (Cyan Fluorescent Protein) were obtained every 25 seconds. YFP (Yellow Fluorescent Protein) and mCherry emissions were also obtained as transfection controls.

### SDS-PAGE and Western Blot

CHO-K1 cells were treated as indicated and lysed with RIPA Buffer (150 mM NaCl, 1% Triton x-100, 50 mM Tris pH 7.5, 1% Sodium Deoxycholate, 0.1% SDS, 2 mM EDTA, 50 mM NaF). Bradford assays were performed to determine the protein concentration in the obtained samples. Samples were run in polyacrylamide gels and transferred to PVDF membranes (BioRad). Membranes were blocked during 30 minutes with a blocking solution (10% BSA, 0.05% Tween 20 in PBS) and washed twice with 0.05% Tween 20 in PBS. Afterward, they were incubated with specific antibodies diluted in 0.05% Tween 20 in PBS Tween: 1 μg/ml rabbit polyclonal anti-LC3 (Sigma-Aldrich), or 0.1 μg/ml rabbit monoclonal anti-actin (Developmental Studies Hybridoma Bank, University of Iowa, USA) overnight at 4°C. Membranes were incubated with secondary antibodies conjugated with peroxidase (Sigma-Aldrich). Detection of immunoreactive bands was performed by chemiluminescence in a FluorChem Q imaging system (Protein Simple).

### Colony Forming Units (CFU) Assay

CHO-K1 cells were transfected with GFP empty vector (GFPv) or GFP-PKCα. Cells were subsequently infected for 2, 3 or 4 h with *S. aureus* wt. At 1 hour post infection (h.p.i.), cells were washed 3 times with 1X PBS, and incubated with gentamycin (100 μg/ml) for 30 minutes to eliminate all extracellular bacteria. After the infection period, cells were washed 3 times with 1X PBS, and once with HBSS. Afterward, cells were lysed with sterile distilled water for 10 minutes at room temperature. Cells were collected using a scraper, and lysates were diluted in 1X PBS, cultured in Brain Heart Infusion agar and incubated for 12 hours at 37°C. Colonies were counted in plates which showed between 50-100 visible colonies.

### Statistical Analysis

All the data are shown as the mean ± standard error of the mean (SEM) and analyzed with GraphPad Prism version 5.01 (GraphPad Software Inc.) using the Student’s t test. Figures shown are representative of ≥3 experiments.

## Results

### Diacylglycerol Is Present in the *S. aureus*-Containing Phagosomes

**Figure 1:**
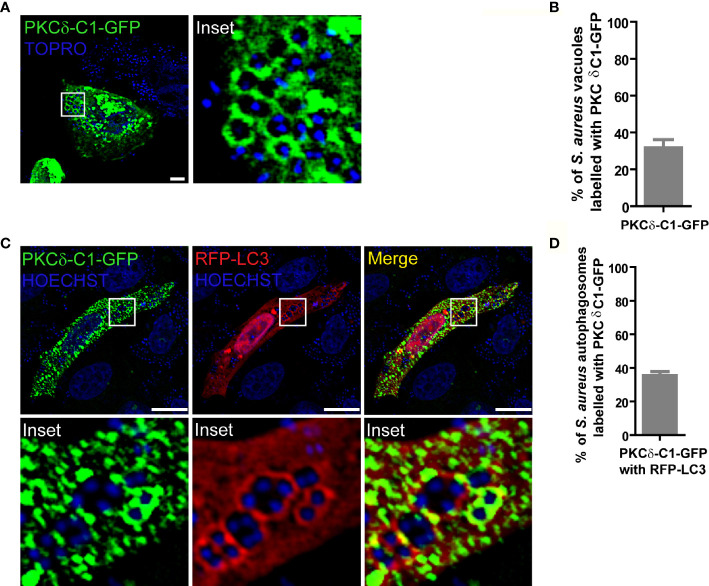
DAG is present in the *S. aureus* phagosomal membranes. **(A)** Confocal microscopy images of CHO cells overexpressing PKCδ-C1-GFP. Cells were infected with *S. aureus* wt for 4 hours as indicated under Material and Methods. Bacteria were labelled with Topro (shown in blue). Images are representative of three independent experiments. Bar: 10 μm. **(B)** Quantification of *S. aureus* vacuoles labelled with PKCδ-C1-GFP. Data are the mean ± SEM of three independent experiments. **(C)** Confocal microscopy images of CHO cells overexpressing PKCδ-C1-GFP together with RFP-LC3 and infected with *S. aureus* wt for 4 hours. Bacteria were labelled with Hoechst (shown in blue). Bar: 10 μm. Images are representative of three independent experiments. **(D)** Quantification of *S. aureus* vacuoles labelled with PKCδ-C1-GFP and RFP-LC3. Data are the mean ± SEM of three independent experiments.

Since DAG is required for activation of most members of the PKC family, we first asked whether the surface of the phagosomal membrane was conducive to PKC activation by assessing whether DAG was located in the *S. aureus* phagosomal membrane. CHO cells were transfected with the DAG sensor, PKCδ-C1-GFP, which consists of the C1 domain of PKCδ fused to green fluorescent protein (GFP). Cells were infected with *S. aureus* wild type (wt, labeled with Topro, shown in blue) and at 4 h.p.i. were fixed and processed for analysis by confocal microscopy. As depicted in **Figure 1**, DAG was clearly present in approximately 40% of the phagosomal membranes containing *S. aureus* (**Figures 1A, B** and **Supplementary Figure 1A**).

Because it has been shown that internalized *S. aureus* resides in autophagosomes (17), we next asked whether DAG was also present in these autophagosomal membranes. For this purpose, CHO cells were cotransfected with the DAG sensor and the autophagic protein LC3 tagged with red fluorescent protein (RFP-LC3). Following infection with *S. aureus* wt, examination by confocal microscopy revealed that approximately 40% of the *S. aureus*-containing autophagosomes, characterized by the presence of LC3 in their membranes, were also labelled with the DAG probe (**Figures 1C, D** and **Supplementary Figure 1B**). These results demonstrate that the second messenger DAG is part of the vacuolar membranes where *S. aureus* resides.

### PKCα Is Recruited to the *S. aureus*-Containing Phagosome

**Figure 2:**
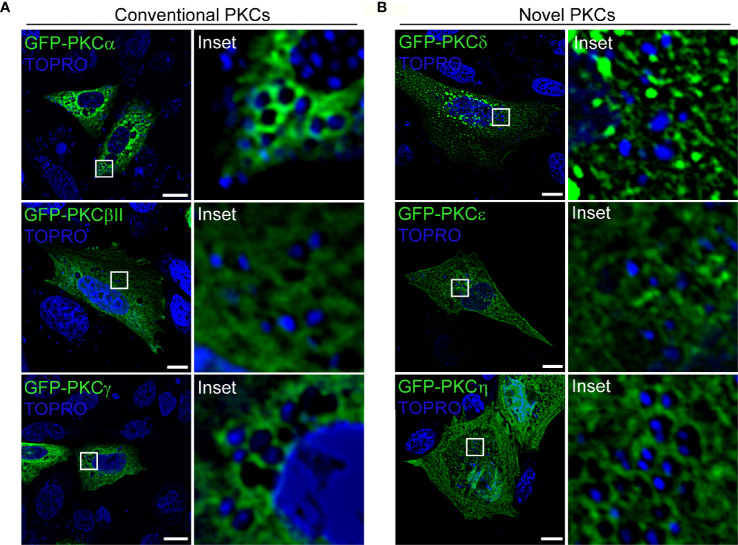
PKCα is recruited to *S. aureus* phagosomes. **(A)** Confocal microscopy images of CHO cells overexpressing each of the three members of the conventional PKCs family: GFP-PKCα, GFP-PKCβII, GFP-PKCγ. Cells were infected with *S. aureus* wt for 4 hours. Bacteria were labelled with Topro (shown in blue). Bar: 10 μm. **(B)** Confocal microscopy images of CHO cells transfected with members of the novel PKC family: GFP-PKCδ, GFP-PKCϵ or GFP-PKCη. Cells were infected with *S. aureus* wt for 4 hours. Bacteria were labelled with Topro (shown in blue). Bar: 10 μm. Figures are representative of five independent experiments.

We next assessed whether the DAG-dependent PKC isozymes (conventional and novel family members) associated with phagosomes harboring *S. aureus*. For this purpose, CHO cells were transfected with the conventional PKC isozymes GFP-PKCα, GFP-PKCβII or GFP-PKCγ (**Figure 2A**), or with the novel PKC isozymes GFP-PKCδ, GFP-PKCϵ or GFP-PKCη (**Figure 2B**). Cells were infected with *S. aureus* wt and, at 4 h.p.i., were analyzed by confocal microscopy. As shown in **Figure 2** (**Supplementary Figure 2A**), we observed that only the conventional isozyme PKCα was clearly recruited to the *S. aureus-*containing phagosomes. To confirm that the recruitment of the kinase was in fact to the phagosomal membranes containing *S. aureus*, GFP-PKCα was cotransfected with RFP-LAMP1, a late endosome marker, which has been proven to be present in the phagosomal membranes containing *S. aureus* (14). Cells were infected with *S. aureus* wt and at 4 h.p.i., confocal microscopy analysis showed that PKCα colocalized with LAMP1 at the membranes of phagosomes containing *S. aureus* (**Supplementary Figures 2B** and **2C**).

### PKCα Recruitment to *S. aureus* Phagosomes Does Not Depend on DAG

**Figure 3:**
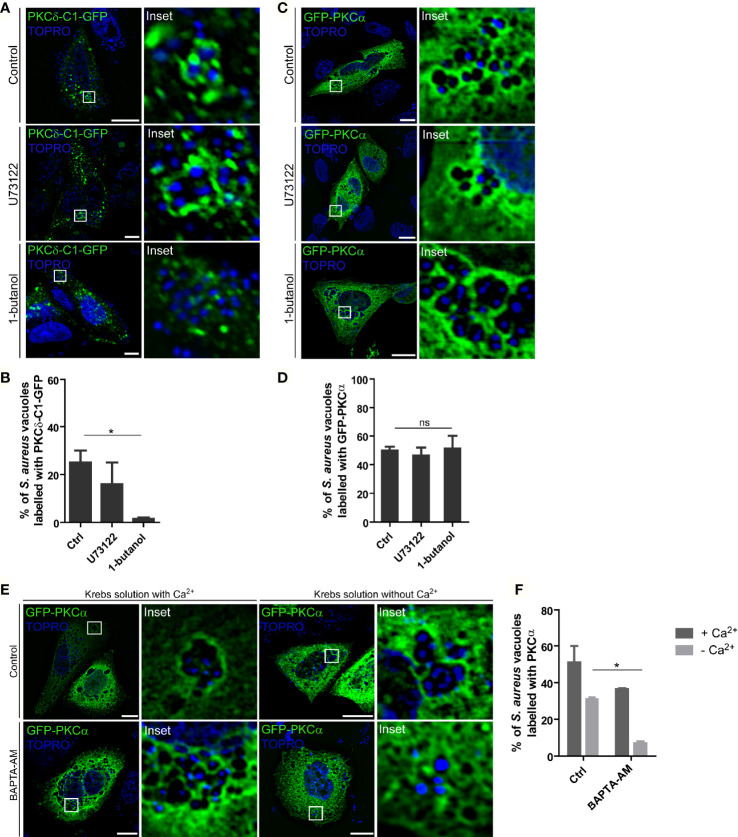
PKCα recruitment to the *S. aureus* phagosomes is independent of DAG but depends on Ca^2+^. **(A)** Confocal microscopy images of CHO cells overexpressing PKCδ-C1-GFP and treated with the PLC inhibitor, U73122 (1.5 µM), or the PLD inhibitor, 1-butanol (0.3% v/v), and infected for 4 hours with *S. aureus* wt. Bacteria were labelled with Topro (depicted in blue). Bar: 10µm. Images are representative of three independent experiments. **(B)** Quantification of *S. aureus* vacuoles decorated with PKCδ-C1-GFP. Data are the mean ± SEM of three independent experiments. *p ≤ 0.01. **(C)** Confocal microscopy images of CHO cells overexpressing GFP-PKCα and treated with the PLC inhibitor, U73122 (1.5 µM) and the PLD inhibitor, 1-butanol (0.3% v/v), and infected for 4 hours with *S. aureus* wt. Bacteria were labelled with Topro (shown in blue). Images are representative of three independent experiments. Bar: 10 μm. **(D)** Quantification of *S. aureus* vacuoles recruiting GFP-PKCα. Data are the mean ± SEM of three independent experiments. **(E)** Confocal microscopy images of CHO cells overexpressing GFP-PKCα treated with 10 μM BAPTA-AM in Krebs solution with (2.5 mM) or without Ca^2+^ and infected with *S. aureus* wt for 4 hours. Bacteria were labelled with Topro (shown in blue). Bar: 10 μm. Images are representative of three independent experiments. **(F)** Quantification of *S. aureus* vacuoles recruiting GFP-PKCα. Data are the mean ± SEM of three independent experiments. *p ≤ 0.05.

To assess whether the DAG present in *S. aureus* phagosomal membranes was necessary to anchor PKCα to these compartments, we examined the effect of DAG synthesis inhibition on PKCα localization to the *S. aureus* phagosomal membranes. CHO cells were transfected with PKCδ-C1-GFP and subsequently treated with inhibitors of DAG synthesis. First, the enzyme phospholipase C (PLC) which generates DAG by the hydrolysis of PIP_2_ (27) was inhibited using U73122. Treatment of *S. aureus* infected cells with the inhibitor U73122 had no significant effect on the amount of DAG present in the *S. aureus* phagosomal membranes (**Figures 3A, B**). Second, cells were treated with 1-butanol to inhibit phospholipase D (PLD). This enzyme catalyzes the hydrolysis of phosphatidylcholine to produce phosphatidic acid which is, in turn, converted into DAG by the action of the phosphatidic acid phosphatase (27). In contrast to the PLC inhibitor, treatment of *S. aureus*-infected cells with the PLD inhibitor caused a decrease in the amount of DAG present in phagosomal membranes (**Figures 3A, B**). These data revealed that PLD was the major pathway responsible for the production of DAG in the *S. aureus*-containing phagosomes.

Once we determined that the inhibition of PLD caused a marked decrease in the presence of DAG in the phagosomal membranes, we asked whether suppressing PLD activity altered the recruitment of PKCα. CHO cells were transiently transfected with GFP-PKCα, treated with either the PLC or PLD inhibitors, and infected with *S. aureus* wt. After 4 h.p.i., cells were fixed and analyzed by confocal microscopy. Surprisingly, as shown in **Figures 3C, D**, there was no change in the presence of the kinase at the *S. aureus*-containing phagosomes. Thus, we concluded that PKCα is recruited to the phagosomal membranes in a DAG-independent manner.

Because PKCα activity and membrane localization is Ca^2+^-dependent, we next explored the dependence on Ca^2+^ in recruiting PKCα to *S. aureus*-containing phagosomes. CHO cells overexpressing GFP-PKCα were treated with the Ca^2+^ chelator BAPTA-AM. As we have previously described, *S. aureus* secretes cytotoxins that are able to generate pores in the plasma membrane allowing the entry of extracellular Ca^2+^ into the cellular cytosol, therefore, we assessed Ca^2+^ chelation in Krebs solution with or without Ca^2+^. In both conditions, cells were infected with *S. aureus* wt and after 4 h.p.i. they were analyzed by confocal microscopy. As shown in **Figures 3E, F**, cells treated with BAPTA-AM in a medium without Ca^2+^ showed a significant decrease of PKCα recruitment to the phagosomal membranes, indicating that this second messenger is required for the translocation and binding of PKCα to the *S. aureus* phagosomes.

### PKCα Recruitment Depends on *S. aureus* α-hemolysin

**Figure 4:**
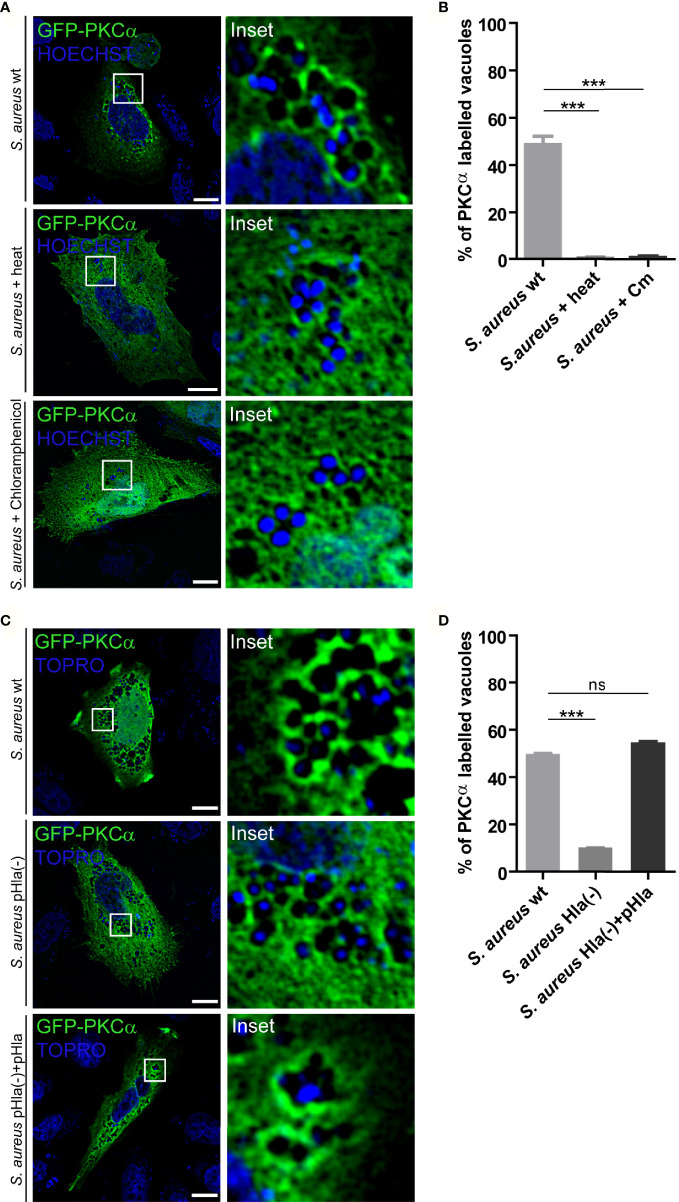
PKCα recruitment to *S. aureus* phagosomes depends on α-hemolysin. **(A)** Confocal microscopy images of CHO cells overexpressing GFP-PKCα, and then infected for 4 hours with *S. aureus* wt, *S. aureus* wt killed by heat or *S. aureus* wt treated with chloramphenicol. Bacteria were labelled with Hoechst (shown in blue). Bar: 10 μm. Images are representative of four independent experiments. **(B)** Quantification of *S. aureus* vacuoles labelled with GFP-PKCα in cells infected with *S. aureus* wt, *S. aureus* wt heat killed or *S. aureus* wt inactivated by chloramphenicol treatment. Data are the mean ± SEM of four independent experiments. ***p ≤ 0.0001. **(C)** Confocal microscopy images of CHO cells overexpressing GFP-PKCα, and then infected with *S. aureus* wt, *S. aureus* Hla (–), mutant deficient of α-hemolysin or *S. aureus* Hla (–)+pHla, mutant complemented with the α-hemolysin for 4 hours. Bacteria were labelled with Topro (shown in blue). Images are representative of five independent experiments. Bar: 10 μm. **(D)** Quantification of *S. aureus* vacuoles labelled with GFP-PKCα in cells infected with *S. aureus* wt, *S. aureus* Hla (–) or *S. aureus* Hla (–)+pHla. Data are the mean ± SEM of five independent experiments. ***p ≤ 0.0001; ns, non significant.

We next sought to determine whether the observed PKCα recruitment was dependent on *S. aureus* viability. For this purpose, bacteria were first inactivated by incubation at 95°C for 10 minutes, and subsequently internalized by CHO cells overexpressing GFP-PKCα. As shown in **Figures 4A, B**, PKCα was not recruited to heat-inactivated *S. aureus*, indicating that the recruitment of PKCα only occurs when the bacteria enclosed in the vacuoles are alive. Next, we addressed whether bacterial protein synthesis was required for PKCα recruitment. *S. aureus* wt was pre-incubated with the inhibitor of bacterial protein synthesis, chloramphenicol. CHO cells were transfected with GFP-PKCα and infected with these bacteria. PKCα was no longer recruited to *S. aureus* when its protein synthesis was prevented (**Figures 4A, B**), confirming that the synthesis of bacterial products was required for PKCα’s association to the pathogen-containing phagosomes.

During staphylococcal infections, the bacteria produce a large amount of secreted virulence factors such as toxins and enzymes. One of the most important virulence factors secreted by *S. aureus* is α-hemolysin (Hla), a cytotoxin able to generate pores in cellular membranes (28). As we have previously demonstrated, this toxin is required for activation of the autophagic pathway during *S. aureus* cellular invasion (16). Having established that synthesis of bacterial proteins is necessary for the association of PKCα with pathogen-containing phagosomes (**Figures 4A, B**), we next addressed whether Hla was one of the bacterial secreted factors responsible for PKCα recruitment. CHO cells overexpressing GFP-PKCα were infected with *S. aureus* wt, the Hla deficient mutant strain *S. aureus* Hla (–), or the complemented mutant *S. aureus* Hla (–)+pHla, which overexpresses the toxin. The samples were processed and analyzed by confocal microscopy. As depicted in **Figures 4C** and **4D**, cells infected with the Hla-deficient mutant showed a marked decrease in the recruitment of PKCα to the bacterial phagosomes. PKCα association was restored to values similar to those observed upon *S. aureus* wt infection when the cells were infected with the complemented strain *S. aureus* Hla (–)+pHla. Taken together, these results indicate that the bacterial production of Hla was critical for the recruitment of PKCα to the phagosomes harboring *S. aureus*.

### *S. aureus* Secreted Factors Activate PKCα

**Figure 5:**
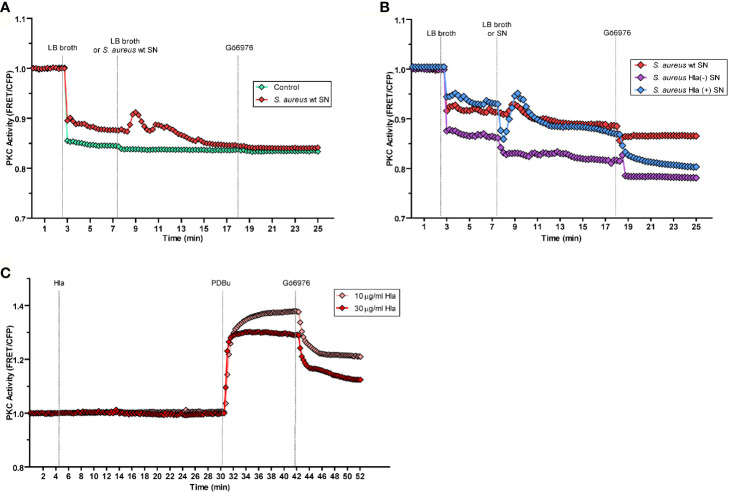
PKCα is activated by the virulence factors secreted by *S. aureus*. By the use of a genetically encoded biosensor, the C kinase activity reporter (CKAR2), PKC activity was assessed as indicated in Material and Methods. **(A)** CHO cells co-expressing CKAR2 and mCherry-PKCα were treated with LB broth to set a baseline. After 5 minutes of stable baseline, they were treated with *S. aureus* wt culture supernatant (red line) and then treated with the PKC inhibitor Gö6976 (1 µM). The control condition (green line) corresponds to LB broth. **(B)** CHO cells co-expressing CKAR and mCherry-PKCα were treated with LB broth to settle a baseline, then, after 5 minutes they were treated with *S. aureus* wt (red line), *S. aureus* Hla (–) (blue line) or *S. aureus* Hla (–)+pHla (violet line) culture supernatants and then treated with the inhibitor Gö6976 (1 µM). **(C)** CHO cells co-overexpressing CKAR2 and mCherry-PKCα were treated with 10 µg/ml (pink line) or 30 µg/ml (red line) of α-hemolysin pure protein. After 25 minutes, cells were treated with the PKC agonist PDBu (200 nM) and 12 minutes afterward, the PKC inhibitor Gö6976 (1 µM) was added. Data are the mean of three independent experiments.

Having established that PKCα was recruited to *S. aureus*-containing phagosomes by a mechanism that depended on the secreted toxin α-hemolysin, we next examined whether bacterial factors were able to activate PKCα. To this end, we used a genetically encoded C Kinase Activity Reporter (CKAR2), which consists of a PKC specific substrate flanked by mCerulean and a yellow fluorescent protein (YFP) (29). Phosphorylation of the reporter by PKC results in a change in the fluorescence resonance energy transfer (FRET) that functions as a read out for activity of the kinase (30). CHO cells cotransfected with CKAR2 and mCherry-PKCα, were treated with *S. aureus* culture supernatants, in order to stimulate cells with those virulence factors secreted by the bacterium. Since the LB broth used to grow bacteria is yellow and interferes with the CFP emission, in all the experiments cells were first treated with LB broth to establish a new baseline and then stimulated with *S. aureus* culture supernatants. Following stimulation with *S. aureus* supernatant, cells were treated with the PKC inhibitor Gö6976, which inhibits conventional PKC isozymes (31). Treatment of cells with the culture supernatant of *S. aureus* wt (red line), but not LB broth alone (green line) resulted in a transient activation of PKC, as assessed by the increase in FRET ratio (**Figure 5A**). These data suggested that factors secreted by *S. aureus* activate PKCα.

We next addressed whether specifically α-hemolysin in the *S. aureus* supernatant was responsible for the observed PKCα activation. Cells overexpressing mCherry-PKCα and CKAR2 were treated with the supernatants from cultures of *S. aureus* wt, *S. aureus* Hla (–), or *S. aureus* Hla (–)+pHla. Whereas treatment of cells with *S. aureus* wt culture supernatant (red line) caused PKC activation, supernatant from the Hla-deficient mutant strain (violet line) did not cause activation of PKC (**Figure 5B**). As expected, activation was restored upon treatment of cells with the supernatant of the mutant complemented with the toxin, *S. aureus* Hla (–)+pHla (blue line) (**Figure 5B**). Indeed, the *S. aureus* Hla (–)+pHla’s supernatant caused an even greater PKC activation peak than the one produced by the wild type strain. Thus, we concluded that the secreted α-hemolysin is necessary for PKCα activation.

Next, in order to determine whether the α-hemolysin itself was able to activate PKCα, CHO cells cotransfected with mCherry-PKCα and CKAR2 were stimulated with two different concentrations of purified toxin, 10 µg/ml and 30 µg/ml. Neither concentration induced activation of the kinase (**Figure 5C**). To confirm that the system was active, cells were stimulated with the phorbol ester PDBu, a PKC agonist, and the expected activation peak was observed. Thus, α-hemolysin was necessary, but not sufficient, for the activation of PKCα. These data suggest that the presence of other virulence factors secreted by the bacterium likely contribute to the activation of PKCα.

### PKCα Inhibits the Autophagy Induced by S*. aureus* Invasion

**Figure 6:**
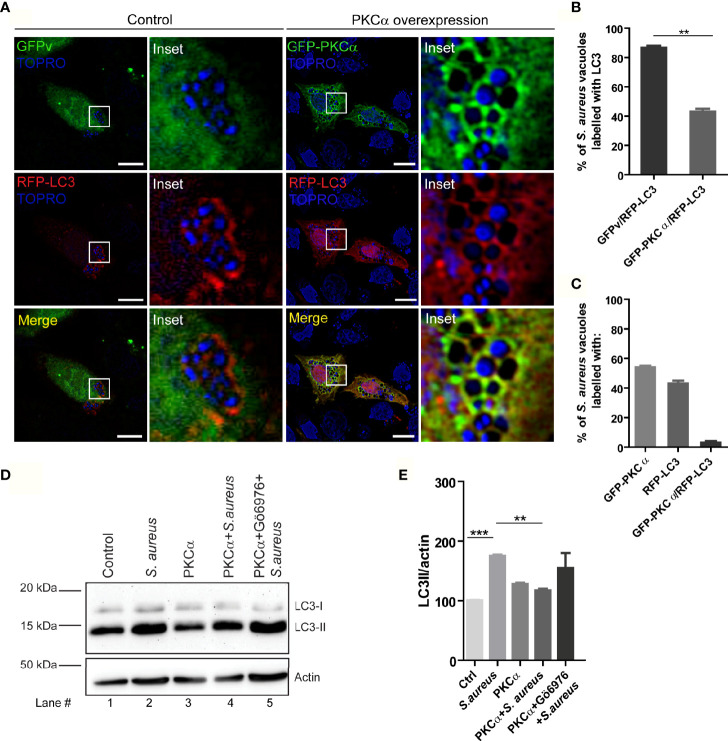
PKCα inhibits autophagy induced by *S. aureus*. **(A)** Confocal microscopy images of CHO cells co-overexpressing GFP-PKCα or GFP empty vector (GFPv) and RFP-LC3 and infected for 4 hours with *S. aureus* wt. Bacteria were labelled with Topro, shown in blue. Bar: 10µm. Images are representative of five independent experiments. **(B)** Quantification of *S. aureus* vacuoles recruiting RFP-LC3. **p ≤ 0.001. **(C)** Quantification of *S. aureus* vacuoles recruiting GFP-PKCα, RFP-LC3 or both. Data are the mean ± SEM of five independent experiments. **(D)** Image of a Western blot analysis corresponding to a membrane incubated with specific antibodies against LC3 and actin (as a loading control), of cell lysates obtained from CHO cells subjected to the following conditions: lane 1, control; lane 2, infected with *S. aureus* wt for 4 hours; lane 3, transfected with mCherry-PKCα; lane 4, transfected with mCherry-PKCα and infected with *S. aureus* wt for 4 hours; line 5, transfected with mCherry-PKCα, treated with the PKC inhibitor Gö6976 (250 nM) and infected with *S. aureus* wt for 4 hours. The figure is representative of four independent experiments. **(E)** Quantification of the Western blot bands intensities with ImageJ. Data are the mean ± SEM of four independent experiments **p ≤ 0.001, ***p ≤ 0.0001.

After demonstrating that the conventional isozyme PKCα is recruited to the phagosomes where *S. aureus* resides during its invasion, and that the secreted factors produced by the bacterium activate the kinase, we next examined whether this enzyme regulates the autophagic response that is induced during infection. First, we analyzed whether PKCα presence in phagosomes affected the recruitment of the autophagic protein LC3 to the phagosomal membrane. Cells were cotransfected with RFP-LC3 and GFP-PKCα, or RFP-LC3 and GFP empty vector as a control, infected with *S. aureus* wt and, after 4 hours, cells were fixed and analyzed by confocal microscopy. Surprisingly, the overexpression of PKCα caused around 40% decrease in the recruitment of the autophagic protein LC3 to the phagosomal membranes (**Figures 6A, B**). We also observed that PKCα and LC3 did not colocalize at the phagosomal membranes, but rather they were mutually exclusive: phagosomes in which PKCα was present had no detectable LC3 and vice versa (**Figure 6C**).

We next examined whether PKCα modulated autophagy by assessing one of the earliest events in autophagy: processing of the autophagic protein LC3 from the cytoplasmic form LC3-I to the lipidated form LC3-II, which is able to bind to autophagosomal membranes. The conversion of LC3-I to LC3-II as detected by Western blot can be used to measure the activation of autophagy. CHO cells were treated in the following conditions: i) control cells; ii) infected with *S. aureus* wt for 4 hours; iii) transfected with mCherry-PKCα; iv) transfected with mCherry-PKCα and infected with *S. aureus* wt; v) transfected with mCherry-PKCα, treated with the inhibitor Gö6976 and infected with *S. aureus* wt. Western blot analysis of cell lysates with a specific antibody for LC3 revealed that, as previously reported (19), LC3-II levels increased when the cells were infected with *S. aureus* wt (**Figures 6D, E**, lane 2). Interestingly, we found that when cells were transfected with PKCα and subsequently infected with *S. aureus*, LC3-II levels decreased significantly (**Figures 6D, E**, lane 4) compared to only infection and no kinase overexpression. Also, we observed that in cells treated with the inhibitor Gö6976 and infected with *S. aureus*, LC3-II levels were reestablished (**Figures 6D, E**, lane 5). Thus, PKCα overexpression results in reduced association of LC3 protein with phagosomes containing bacteria as assessed by confocal microscopy, and reduced LC3-II levels, as assessed by Western blot. Taken together, these results indicate that PKCα is able to inhibit the autophagic response induced during *S. aureus* infection.

### PKCα Inhibits *S. aureus* Intracellular Replication

**Figure 7:**
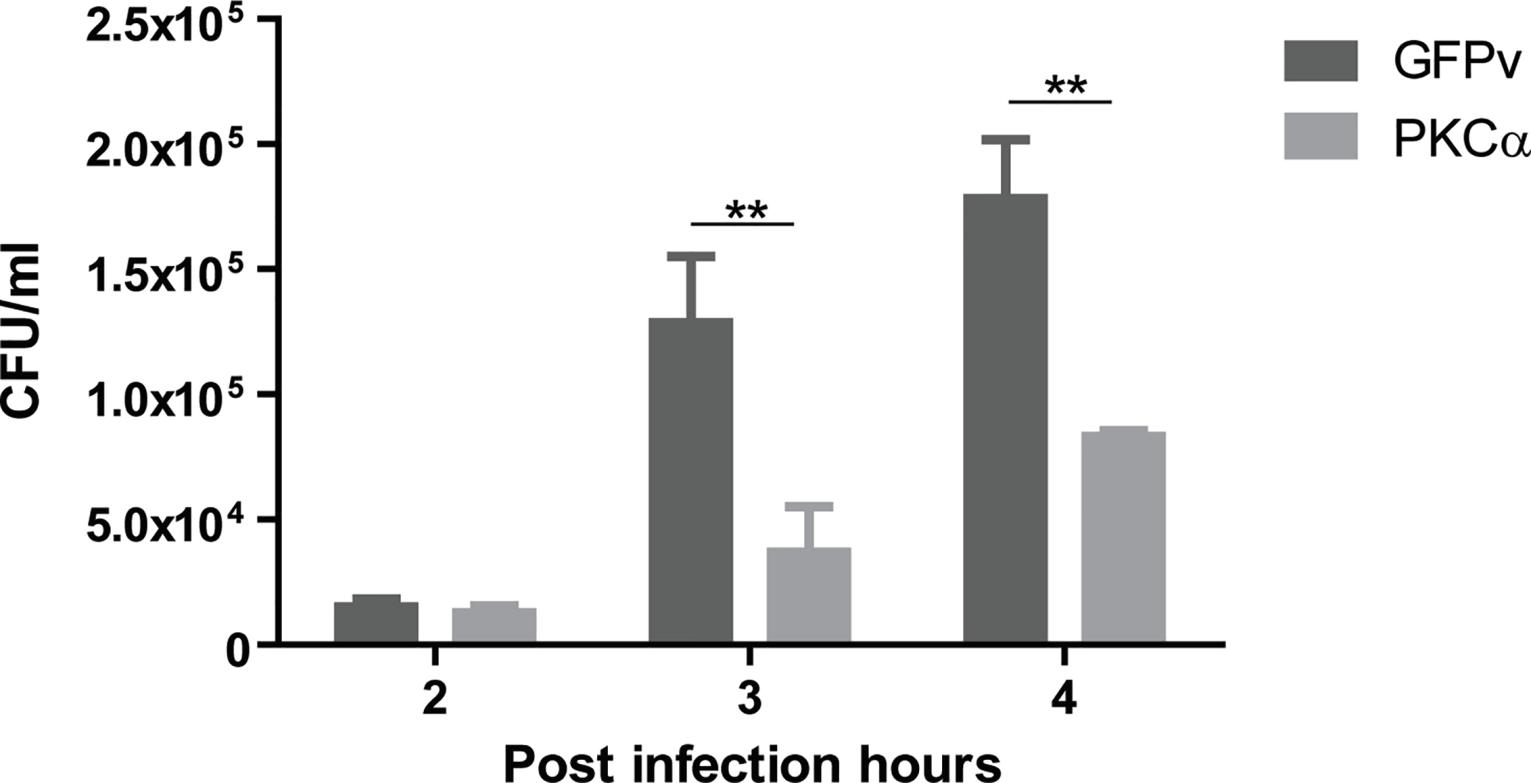
PKCα decreases intracellular replication of *S. aureus*. Colony forming units (CFU) quantification (see Materials and Methods) of CHO cells overexpressing GFP-PKCα or GFPv and infected for 4 hours with *S. aureus* wt. Data are the mean ± SEM of three independent experiments. **p ≤ 0.001.

*S. aureus* is one of the pathogen microorganisms that modulates the autophagic pathway for its own benefit, utilizing the autophagosomes as a protective niche, where it actively replicates before escaping toward the cytoplasm (32). Given that PKCα inhibited the autophagic response induced by *S. aureus*, we reasoned that the ability of *S. aureus* to replicate in the interior of cells might be regulated by PKCα. To assess this, CHO cells were transfected with GFP-PKCα or GFP empty vector as a control, infected with *S. aureus* wt and lysed after 2, 3 or 4 hours. Samples were cultured in Brain Hearth Infusion Agar to allow quantification of bacterial colonies. As depicted in **Figure 7**, PKCα expression caused a significant decrease in the number of Colony Forming Units (CFU) compared to cells transfected with the empty vector. Therefore, we concluded that the overexpression of PKCα significantly impairs *S. aureus* intracellular replication.

## Discussion

*S. aureus* is a pathogen responsible for a broad range of diseases that vary from local controlled infections to life-threatening systemic infections. The understanding of staphylococcal infections has gained importance given the high antibiotic resistance that the bacterium has developed in recent years. It is crucial to find new ways of impairing the ability of this bacterium to replicate in the interior of cells in order to modulate the damage that it causes.

During bacterial infection, several mechanisms are triggered. It is a battle between the survival mechanisms of the bacteria and the countless signaling pathways that the cells activate in order to avoid bacterial replication and promote their removal from the host cell. Xenophagy is meant to be a degradation pathway that contributes to the elimination of foreign pathogens, but *S. aureus* utilizes the autophagosomes as a replicative niche (33). Here, we have unraveled one novel signaling pathway that can be used to prevent *S. aureus* from taking advantage of the autophagic pathway for its replication. In this study, we have demonstrated that the overexpression of PKCα during *S. aureus* infection causes the recruitment of this kinase to the phagosomal membranes, hampering the autophagic response induced by the invasion of the bacterium, most likely by phosphorylation of the autophagy protein LC3 (34). Moreover, we have shown that overexpression of PKCα generates an important decrease in the intracellular replication of *S. aureus* in epithelial cells.

**Figure 8:**
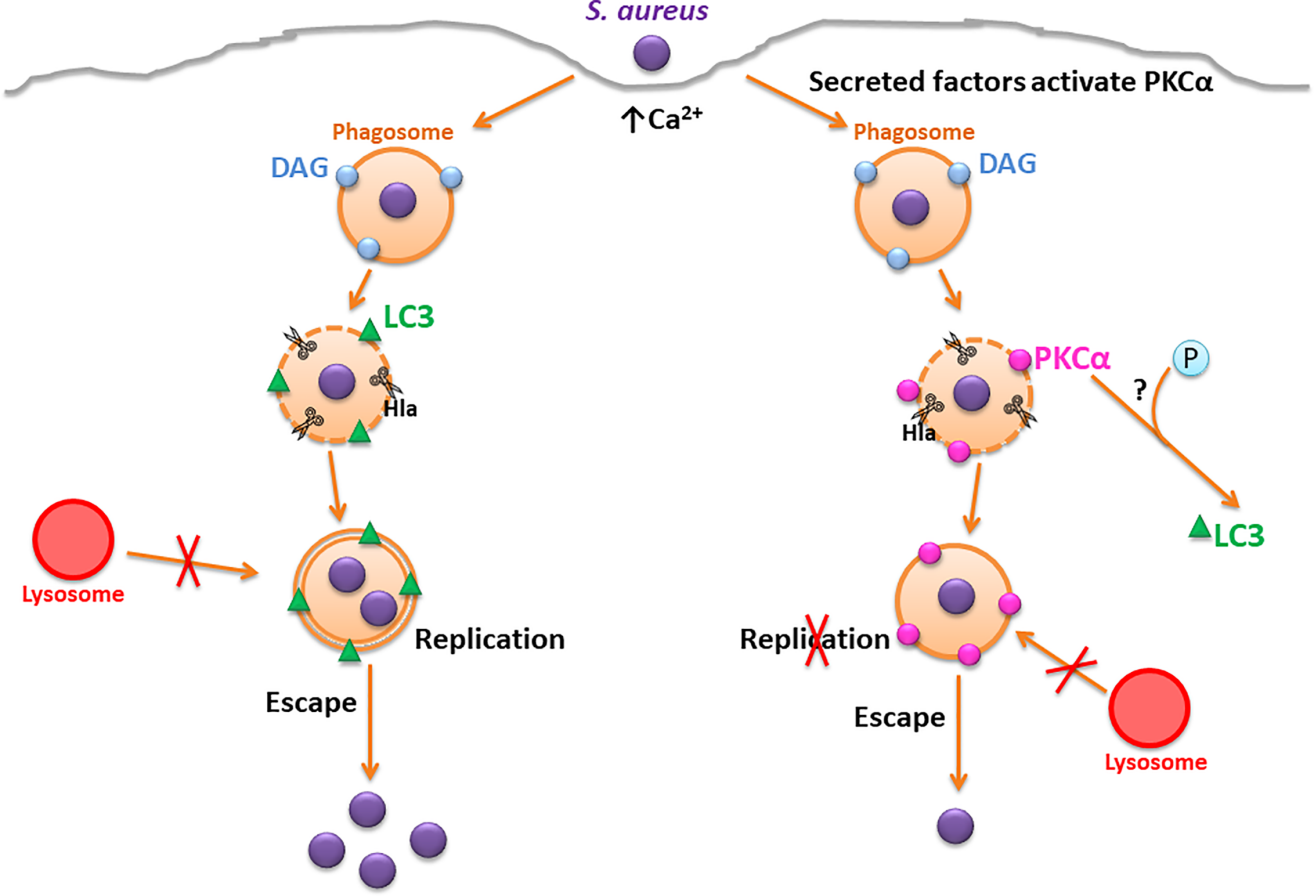
Proposed model. On the left, the canonical *S. aureus* intracellular traffic is depicted, where Hla causes the disruption of the phagosomal membrane and the activation of autophagic response. *S. aureus* replicates in the interior of autophagosomes where it resides and finally, escapes to the cytoplasm. On the right, the overexpression of PKCα causes a disruption in the regular *S. aureus* traffic. Hla causes the recruitment of PKCα to the phagosomal membranes, which inhibits autophagy and impairs *S. aureus* intracellular replication.

Our proposed model suggests that during *S. aureus* infection, after internalization, the bacteria follow the previously described intracellular pathway, residing in a phagosome whose membrane is disrupted by Hla causing the recruitment of the autophagic protein LC3. The bacteria use the double membrane autophagosomes to actively replicate and subsequently, escape toward the cytoplasm. However, when PKCα is overexpressed during *S. aureus* infection, the intracellular pathway that the bacteria transit is altered: the action of the bacterial Hla and other virulence factors secreted by *S. aureus* cause the activation of PKCα. Then, the kinase is recruited to the phagosomal membranes by action of the Hla. We hypothesize that PKCα phosphorylates LC3, preventing its association with the membranes of the compartments containing the bacteria, causing the inhibition of autophagy and in turn, inhibiting the intracellular replication of *S. aureus* (**Figure 8**).

The lipid DAG is an important second messenger involved in a wide range of signaling pathways. Jongstra-Bilen and collaborators showed an accumulation of DAG in the phagosomal membranes containing opsonized latex beads. A burst of DAG was observed during phagosomal formation by the action of Bruton’s tyrosine kinase (Btk) (35). It has also been reported that the autophagosomes where *Salmonella typhimurium* resides present DAG in their membranes, which is required for the activation of antibacterial autophagy (36). Certain species of *Listeria* and *S. aureus* itself produce a phospholipase C enzyme that is able to generate DAG in the host cells (37). Indeed, *S. aureus* phospholipase C is considered an important virulence factor that contributes to lung injury during staphylococcal infections. In the present results, we have determined that DAG was present in 40% of phagosomes containing *S. aureus* (**Figure 1**), which were also labeled by the autophagic protein LC3 (i.e. autophagic compartments), a similar behavior as the one observed during *S. typhimurium* infection (36).

After screening the members of the PKC family that bind to DAG, we found that the conventional isozyme PKCα is recruited to the membranes of *S. aureus* containing-phagosomes (**Figure 2** and **Supplementary Figure 2**). It is known that PKCϵ is present in the phagosomal membranes of opsonized beads (38); it has also been observed that PKCα is recruited to phagosomes containing latex beads in murine macrophages, an interaction that is crucial for the maturation of those phagosomes (26). However, to the best of our knowledge, our findings report the presence of this kinase in the phagosomes containing live bacteria for the first time.

The activation of conventional PKC isozymes requires the binding of the two second messengers DAG and Ca^2+^ to the C1 and C2 domains respectively (39). When DAG synthesis was inhibited, we observed that despite the lack of DAG in the *S. aureus* phagosomal membranes, PKCα still associated with these membranes (**Figure 3**), suggesting that the binding of the kinase to these compartments is independent of DAG. This behavior has been reported in the past, and it has been shown that PKCα can also be bound to membranes by protein-protein interactions (40, 41). Further experiments would be necessary to identify possible PKCα recruitment molecules to the vacuole membranes harboring *S. aureus*. In contrast, we have demonstrated that Ca^2+^ was required for the recruitment of PKCα to the phagosomal membrane. Eichstaedt and collaborators provided evidence that *S. aureus* alpha toxin (Hla) leads to an increase in the intracellular Ca^2+^ levels in a dose- and time-dependent manner (20). We have also demonstrated that the recruitment of PKCα to the phagosomes containing *S. aureus* is dependent on Hla, because in cells infected with the *S. aureus* mutant strain deficient for Hla, PKCα recruitment was lost (**Figure 4**). Taken together, it is likely that during *S. aureus* invasion, the pores made at the phagosomal membranes by the action of Hla, may cause a localized increase of Ca^2+^ concentration that promotes the association of this kinase to the phagosomes. In addition, we have established that the activation of PKCα needs the presence of Hla, although this toxin is not able to activate PKCα on its own, since other virulence factors produced and secreted by *S. aureus* seem to be required (**Figure 5**). The activation of PKC isozymes during bacterial infections has been described previously: *E. coli* activates PKCϵ during its invasion (23), *Listeria monocytogenes* activates PKC in order to be able to escape from phagosomes (42), and PKCδ is activated during *S. typhimurium* infection in order to activate the antibacterial autophagy (36). However, this is the first report showing that factors secreted by *S. aureus* activate PKCα.

Of note, we established that the overexpression of PKCα causes an inhibition in the autophagy induced by *S. aureus* (**Figure 6**). It has also been shown that PKCα has a role in the regulation of autophagy induced by other stimuli, for example, a pro-autophagy role has been assigned to PKCα in the autophagic response induced by palmitic acid (43). Additionally, it has been shown that PKCα can promote autophagy by mitochondrial disruption and ROS generation (44), but this is the first time that a role in the autophagy triggered by bacterial infection is given to PKCα. In the present report we have shown by confocal microscopy that the overexpression of PKCα during *S. aureus* infection caused a marked decrease in the recruitment of the autophagic protein LC3 to phagosomes containing the bacteria. We have also shown that PKCα negatively regulates the LC3-II levels by Western blot analysis when comparing cells infected with *S. aureus* with cells overexpressing the kinase and infected with the pathogen. Consistent with this, Jiang and collaborators have shown that PKC lead to inhibition of starvation-induced autophagy (34). It is likely that a similar mechanism is triggered during *S. aureus* infection, but further studies are needed to confirm this hypothesis.

We and others have previously demonstrated that transit of *S. aureus via* the autophagic pathway is beneficial for pathogen survival (16, 17, 32). The biological importance of all our findings is the fact that the overexpression of PKCα, through inhibition of autophagy, causes a marked hampering in the intracellular replication of *S. aureus* (**Figure 7**). It has been shown that PKCδ has a similar effect during *S. typhimurium* infection, causing the elimination of the bacterium, but in this case, the effect has been attributed to activation of autophagy (36). In this regard, it is important to note that autophagy is detrimental for *S. typhimurium*, but beneficial for *S. aureus*. Thus, both PKCs are important for bacterial degradation but by distinct mechanisms. It has been shown that PKCα has an important role in controlling infections in macrophages, since the overexpression of a dominant negative mutant of PKCα caused enhanced survival of *Leishmania donovani* and further replication of *Legionella pneumophila* (25). All these findings point to a new important focus in the study of antibacterial mechanisms, where PKCs play an essential role in these processes and deserve further studies as therapeutic targets as an alternative to antibiotic treatments.

## Data Availability Statement

The original contributions presented in the study are included in the article/**Supplementary Material**. Further inquiries can be directed to the corresponding author.

## Author Contributions

MG: methodology and investigation. MG, AN, and MC: writing, review, and editing. MC and AN: funding acquisitions and resources. MC: supervision. All authors contributed to the article and approved the submitted version.

## Funding

This work was supported by PICTs 2013-0305 and 2016-0443 from the ANPCYT and SIIIP 06/J470 (MC), NIH R35 GM122523 (AN). MG was supported by a CONICET (Consejo Nacional de Investigaciones Científicas y Técnicas de Argentina) Ph.D. Fellowship and in part by an IUBMB (International Union of Biochemistry and Molecular Biology) Wood-Whelan Fellowship.

## Conflict of Interest

The authors declare that the research was conducted in the absence of any commercial or financial relationships that could be construed as a potential conflict of interest.
