# Supplementary material for: PKCα Is Recruited to Staphylococcus aureus-Containing Phagosomes and Impairs Bacterial Replication by Inhibition of Autophagy
Source: Front Immunol. 2021 Mar 18;12:662987. doi: 10.3389/fimmu.2021.662987 (PMC8013776; doi:10.3389/fimmu.2021.662987)
Supplement: Supplementary file 1 [file DataSheet_1.docx]

***Supplementary material***

**PKCα is recruited to *Staphylococcus aureus-*containing phagosomes and modulates the autophagic response induced by the bacterium**

**Maria Celeste Gauron, Alexandra C. Newton and María Isabel Colombo***

* **Correspondence:** Corresponding author: María Isabel Colombo. E-mail: [mcolombo@fcm.uncu.edu.ar](mailto:mcolombo@fcm.uncu.edu.ar)

**Supplementary Figures**

**Supplementary Figure 1**


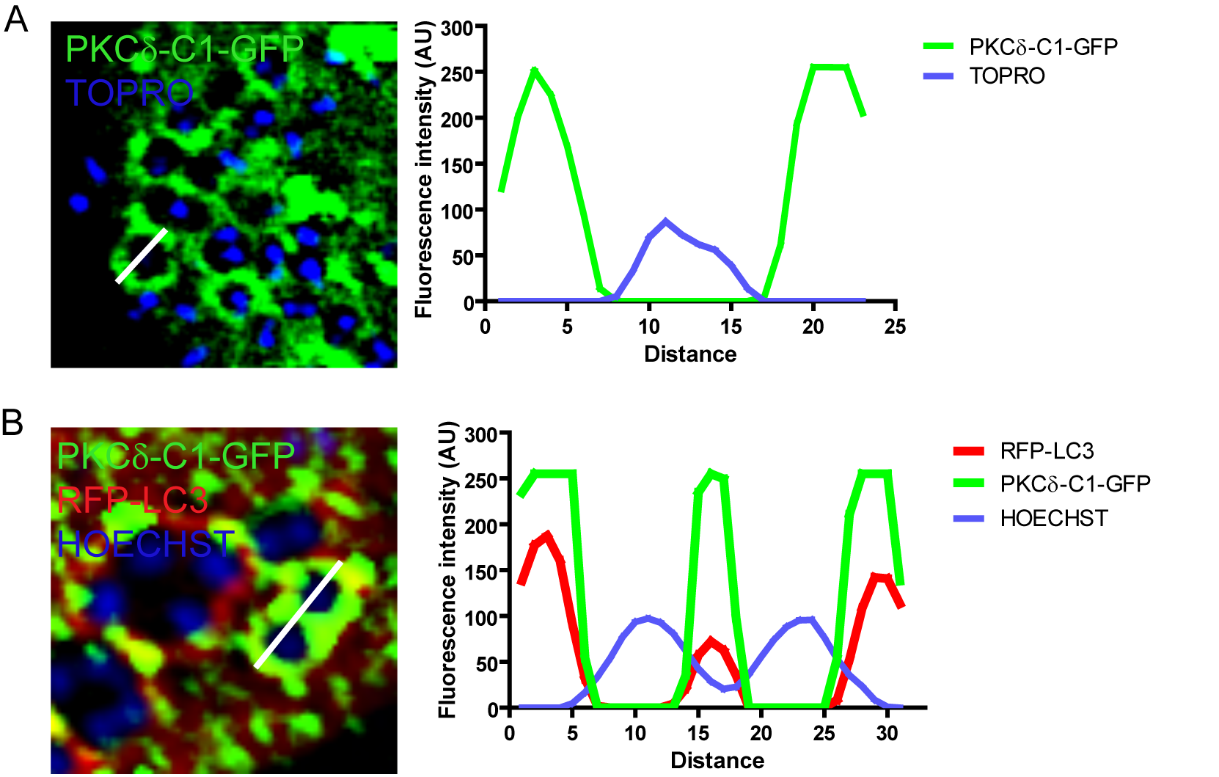


**Supplementary figure 1: DAG is present in the *S. aureus* phagosomal membranes.** **(A)** Fluorescence intensity along the white line depicted in the inset corresponding to Figure 1A. CHO cells overexpressing PKCδ-C1-GFP and infected with *S. aureus* wt (labelled with Topro, showed in blue). **(B)** Fluorescence intensity along the white line depicted in the inset corresponding to Figure 1B. CHO cells overexpressing PKCδ-C1-GFP together with RFP-LC3 and infected with *S. aureus* wt (labelled with Hoechst, showed in blue).

**Supplementary Figure 2**


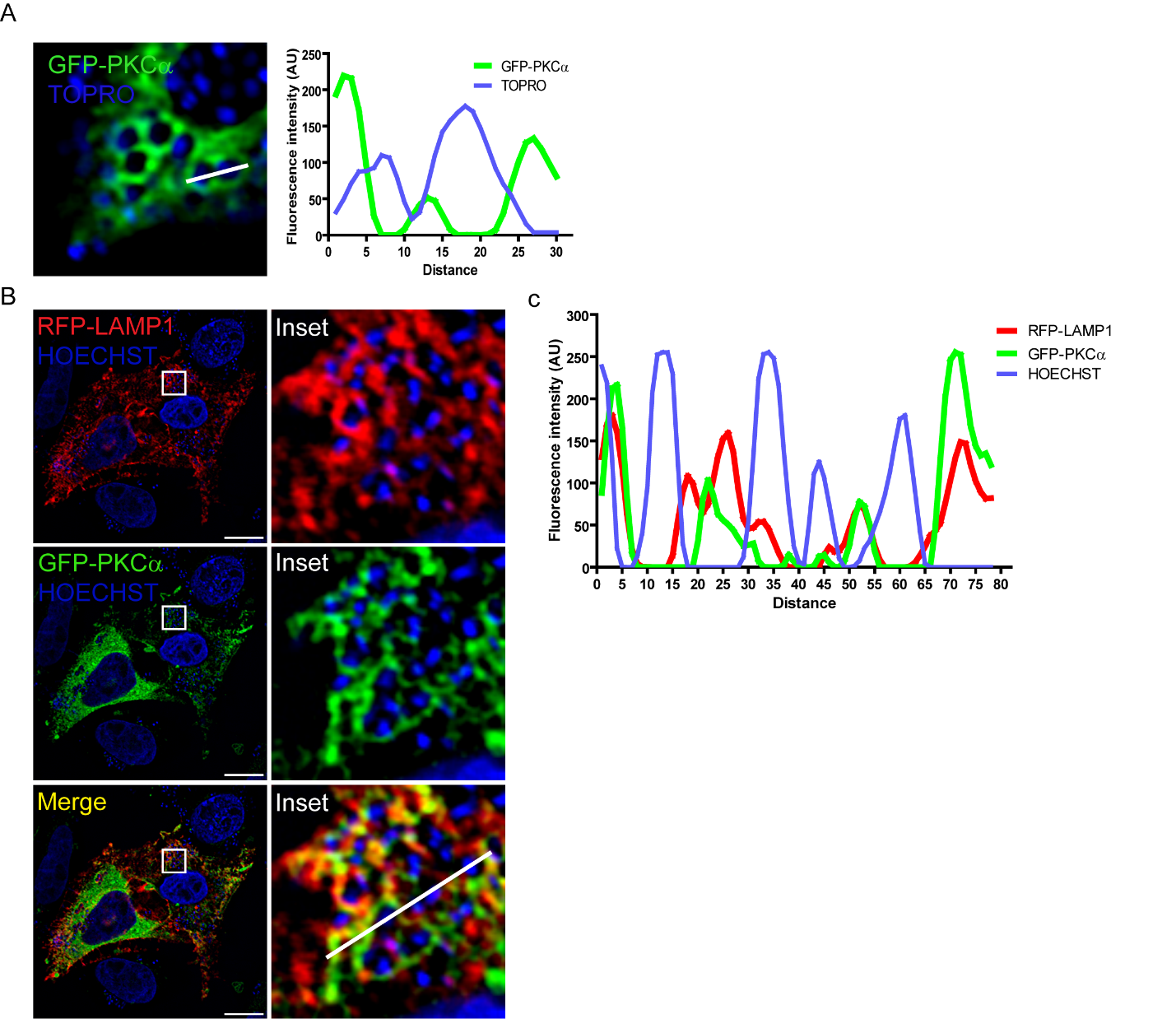


**Supplementary figure 2: PKCα is recruited to *S. aureus* phagosomes. (A)** Fluorescence intensity along the white line depicted in the inset corresponding to Figure 2A. CHO cells overexpressing GFP-PKCα (green) and infected with *S. aureus* wt (labelled with Topro, showed in blue). (**B)** Confocal microscopy images of CHO cells co-overexpressing GFP-PKCα (green) and RFP-LAMP1(red) and infected for 4 hours with *S. aureus* wt. Bacteria were labelled with Topro, shown in blue. Bar: 10µm. **(C)** Fluorescence intensity along the white line depicted in the inset in panel B.
